# Glucose/potassium ratio and a novel combined model for the differential diagnosis of testicular torsion: a retrospective study

**DOI:** 10.1186/s12610-026-00313-5

**Published:** 2026-05-04

**Authors:** Yasin Aktaş, Adem Tunçekin

**Affiliations:** 1 Faculty of Medicine, Department of Urology, Usak University, Fevzi Çakmak, Gazi Boulevard Side Street No: 50, Uşak, 64300 Uşak Centre Turkey; 2 Department of Urology, Uşak Training and Research Hospital, Fevzi Çakmak, Gazi Boulevard Side Street No: 50, Uşak, 64300 Uşak Centre Turkey

**Keywords:** Testicular torsion, Epididymorchitis, Glucose/potassium ratio, Ischaemia-reperfusion injury, Biomarkers, Torsion testiculaire, Epididymo-orchite, Rapport Glucose/Potassium, Lésion Ischémie-Reperfusion, Biomarqueurs

## Abstract

**Background:**

It is challenging to distinguish between testicular torsion (TT) and epididymorchitis (EO) due to their overlapping symptoms. This study evaluated the diagnostic value of the glucose/potassium ratio (GPR) and a combined model for distinguishing between TT, EO, and non-specific acute scrotal pain. This retrospective study included 373 patients, who were divided into three groups: TT (*n* = 103), EO (*n* = 120), and non-specific pain controls (*n* = 150). GPR and systemic inflammatory indices were calculated from admission blood samples. A combined model incorporating independent predictors (age, GPR, and white blood cell (WBC) count) was constructed using binary logistic regression. Diagnostic performance was assessed using a receiver operating characteristic (ROC) curve and multivariate logistic regression analysis.

**Results:**

The median GPR was highest in the TT group (23.6), followed by the EO group (21.0) and the control group (20.4) (*p* < 0.001). A GPR cut-off value of > 21.8 was associated with a 3.52-fold increased risk of testicular torsion (OR: 3.52, 95% CI: 1.97–6.25; *p* < 0.001). Although the GPR demonstrated the greatest accuracy among the individual markers (AUC: 0.738), the combined model produced superior diagnostic results, with an AUC of 0.839 (sensitivity: 75.6%; specificity: 81.5%; positive predictive value (PPV): 60.9%; negative predictive value (NPV): 89.7%). Multivariate analysis identified the following as independent predictors of testicular torsion: younger age (OR: 0.92); higher WBC count (OR: 1.30); and higher GPR levels (OR: 1.14).

**Conclusions:**

GPR appears to be a biomarker that could reflect specific metabolic stress in testicular ischaemia, which is distinct from the inflammatory response in EO. The combined model demonstrated the highest diagnostic accuracy in our cohort, suggesting its potential usefulness as an adjunctive triage tool. However, these findings are preliminary and require external validation in future prospective, multicentre studies before routine clinical implementation.

## Résumé

**Contexte:**

Il est difficile de distinguer une torsion testiculaire (TT) d’une épididymo-orchite (EO) en raison du chevauchement de leurs symptômes. La présente étude a évalué la valeur diagnostique du rapport glucose/potassium (RGP) ainsi qu’un modèle combiné pour distinguer entre TT, EO et douleur scrotale aiguë non spécifique. Cette étude rétrospective a inclus 373 patients, répartis en trois groupes : TT (n = 103), EO (n = 120) et témoins d’une douleur non spécifique (n = 150). Le RGP et les indices inflammatoires systémiques ont été calculés à partir d’échantillons sanguins lors de l’admission. Un modèle combiné incorporant des prédicteurs indépendants (âge, RGP et nombre de globules blancs (GB)) a été construit à l’aide d’une régression logistique binaire. La performance diagnostique a été évaluée à l’aide d’une courbe de caractéristiques de fonctionnement du récepteur (ROC) et d’une analyse de régression logistique multivariée.

**Résultats:**

La valeur médiane du RGP était la plus élevée dans le groupe TT (23,6) suivi par le groupe EO (21) et le groupe témoin (20,4) (p < 0,001). Une valeur seuil du RGP supérieure à 21,8 était associée à un risque accru de torsion testiculaire de 3,5 fois plus grand (OR : 3,52 ; IC à 95 % : 1,97–6,25 ; p < 0,001). Bien que le RGP ait démontré la plus grande précision parmi les marqueurs individuels (AUC : 0,738), le modèle combiné a produit des résultats diagnostiques supérieurs, avec une AUC de 0,839 (sensibilité : 75,6 % ; spécificité : 81,5 % ; valeur prédictive positive : 60,9 % ; valeur prédictive négative : 89,7 %). L’analyse multivariée a identifié les prédicteurs indépendants de la torsion testiculaire : un âge plus jeune (OR : 0,92) ; un nombre plus élevé de GB (OR : 1,30) ; et des niveaux de RGP plus élevés (OR : 1,14).

**Conclusions:**

Le RGP semble être un biomarqueur pouvant refléter un stress métabolique spécifique de l’ischémie testiculaire, ce qui est distinct de la réponse inflammatoire dans l’EO. Le modèle combiné a démontré la plus grande précision diagnostique dans notre cohorte, ce qui suggère son utilité potentielle comme outil de tri complémentaire. Cependant, ces résultats sont préliminaires et nécessitent une validation externe dans de futures études prospectives multicentriques avant une mise en œuvre clinique de routine.

## Background

Testicular torsion (TT) is a urological emergency caused by rotation of the spermatic cord and subsequent ischaemia [1]. It predominantly affects males under 21 years old (incidence: ~15/100,000), and prompt intervention is critical as ischaemia exceeding four to eight hours typically results in irreversible damage and orchiectomy [2, 3].

The primary clinical challenge in acute scrotal pain is the distinction between TT and epididymorchitis (EO), as they share similar symptoms but require opposing management strategies (immediate surgery vs. antibiotics) [4]. Misdiagnosis can result in organ loss or unnecessary surgical exploration [5]. Although Colour Doppler Ultrasonography (CDUS) is the gold standard for diagnosis, its limitations regarding operator dependency and equivocal results necessitate the development of reliable, objective biomarkers [6].

Haematological parameters, including the neutrophil-to-lymphocyte ratio (NLR), platelet-to-lymphocyte ratio (PLR), monocyte count, and systemic inflammation indices, have been utilised in the differential diagnosis of acute scrotal pathologies [7], yet their diagnostic value is limited by low specificity [8]. As EO is inherently an infectious and inflammatory process, it inevitably leads to elevated levels of these inflammatory markers, which are similar to the ischaemia-reperfusion injury observed in TT [9]. This complicates the ability of traditional inflammatory indices to reliably distinguish between ischaemic (TT) and infectious (EO) aetiologies.

Recently, the glucose/potassium ratio (GPR) has emerged as a significant biomarker for predicting disease severity and mortality, particularly in conditions involving acute ischaemia, such as stroke, heart failure, and trauma [10–12]. In acute stress states, elevated catecholamines and cortisol increase blood glucose while decreasing potassium levels [13]. Consequently, GPR may serve as a novel indicator of the body’s stress response and metabolic imbalance, potentially offering a different pathophysiological perspective than pure inflammation markers. We hypothesised that acute ischaemic pain and oxidative stress associated with TT might trigger a more potent sympathoadrenal surge than the gradual inflammatory process of EO.

To our knowledge, the clinical utility of GPR in this context has not been explored before. The aim of this study is therefore to evaluate the diagnostic value of GPR as a metabolic biomarker in comparison with systemic inflammatory indices. Additionally, we aimed to develop a combined diagnostic model to enhance the differential diagnosis of TT, EO, and non-specific scrotal pain.

## Materials and methods

This retrospective observational study involved a total of 373 participants who were treated at our institution between 2020 and 2024. The study protocol was approved by the Uşak University Clinical Research Ethics Committee (approval no. 734-734-07, date 26/06/2025). Due to the retrospective nature of the study, the ethics committee waived the requirement for written informed consent. All patient data were anonymised and de-identified prior to analysis to ensure confidentiality, and the study was conducted in full accordance with the ethical standards of the Declaration of Helsinki.

The study population was categorised into three distinct groups based on the final diagnosis, which was confirmed by clinical evaluation and CDUS. The TT group comprised 103 patients presenting with acute scrotal pain, and the diagnosis was confirmed by the absence of testicular blood flow on CDUS and subsequent surgical findings. The EO group included 120 patients who were diagnosed with epididymitis or epididymo-orchitis based on clinical signs such as swelling, tenderness, and fever, as well as CDUS findings of increased blood flow and epididymal enlargement. The control group comprised 150 patients who presented to the emergency department with acute scrotal pain similar to that experienced by the other groups. TT and EO were definitively excluded in these patients based on physical examination and CDUS findings. This group comprised patients with non-ischaemic, non-infectious, and non-traumatic causes of scrotal pain, specifically categorised as idiopathic scrotal pain.

**Fig. 1:**
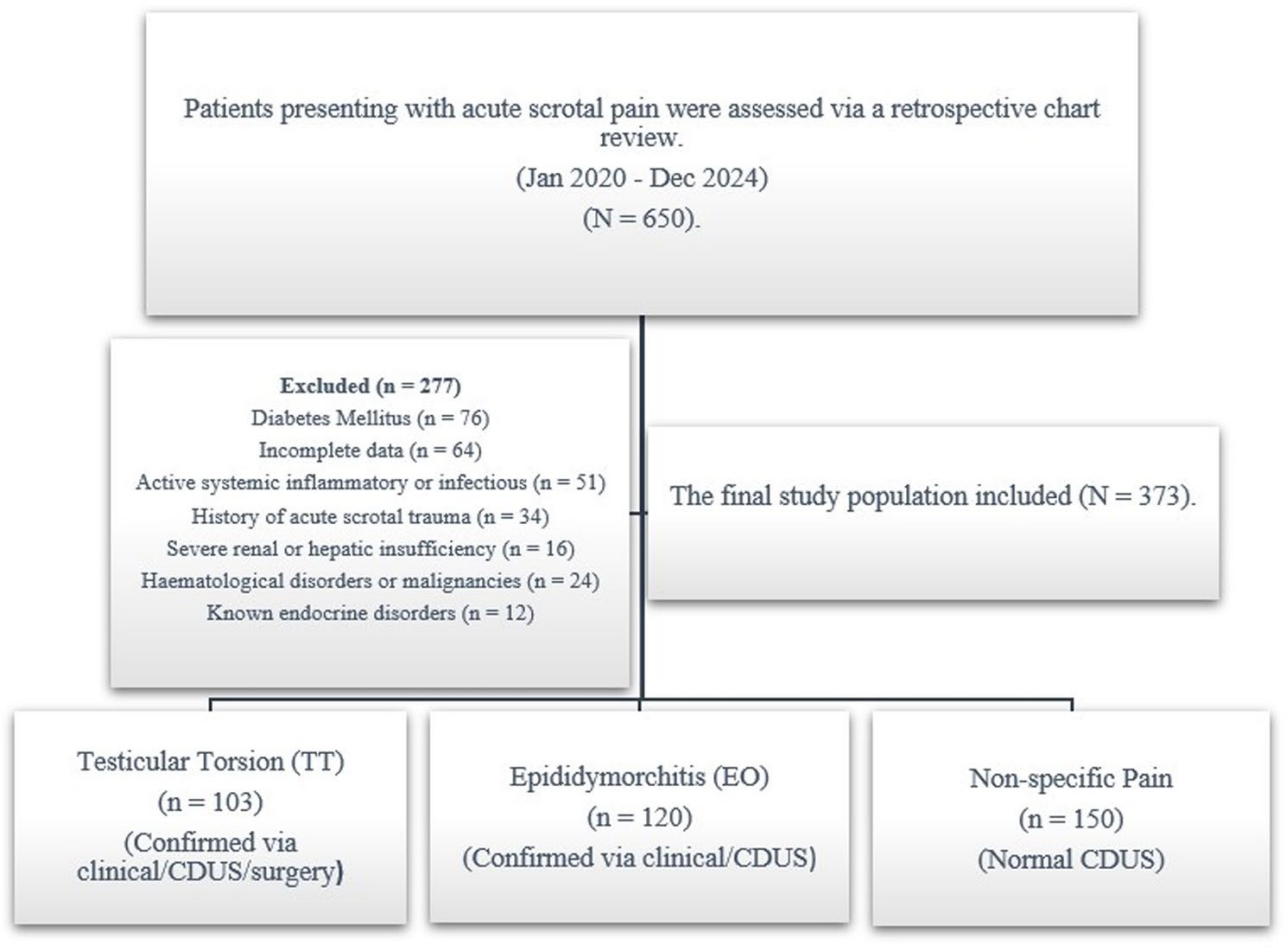
Flow chart of patient selection and study methodology. (Legend: The diagram illustrates the retrospective evaluation of 650 patients with acute scrotal pain. It details the exclusion of 277 patients. It also categorises the remaining 373 patients. These patients are categorised into the following groups: testicular torsion (TT), epididymorchitis (EO), and non-specific scrotal pain. Abbreviations: CDUS: Colour Doppler Ultrasonography; EO: Epididymorchitis; TT: Testicular Torsion.)

The exclusion criteria for all participants included a history of acute scrotal trauma, haematological disorders, malignancies, active systemic inflammatory or infectious diseases (other than EO for the study group), severe renal or hepatic insufficiency, and specific endocrine disorders known to affect glucose or potassium metabolism (e.g., Cushing’s syndrome, adrenal insufficiency, hyperaldosteronism, and uncontrolled thyroid diseases). Patients with diabetes mellitus were excluded to prevent confounding effects on glucose levels, given the study’s focus on the GPR. The detailed patient selection process and attrition rates are illustrated in Fig. 1.

Venous blood samples were collected from the antecubital vein upon initial admission to the emergency department or urology outpatient clinic. Samples for a complete blood count (CBC) were collected in tubes containing dipotassium ethylenediaminetetraacetic acid (K₂EDTA), and samples for biochemical analysis were collected in serum separator tubes. CBC parameters, including neutrophil, lymphocyte, monocyte, eosinophil, and platelet counts, were measured using a Mindray automated haematology analyser (Mindray Bio-Medical Electronics, Shenzhen, China). Serum glucose and potassium levels were analysed using the Alinity clinical chemistry system (Abbott Diagnostics, Abbott Park, IL, USA). Glucose levels were recorded in milligrams per decilitre (mg/dL) and potassium levels in millimoles per litre (mmol/L).

Specific inflammatory indices and ratios were calculated from the results of these laboratory tests. The GPR was calculated by dividing the serum glucose level (mg/dL) by the potassium level (mmol/L). Other indices calculated included the NLR, PLR, monocyte-to-lymphocyte ratio (MLR), systemic immune-inflammation index (SII: NLR × platelet count), systemic inflammation response index (SIRI: NLR × monocyte count), and the aggregate index of systemic inflammation (AISI: NLR × platelet count × monocyte count). Additionally, novel eosinophil-based indices were derived for analysis, including the neutrophil-to-eosinophil ratio (NER), monocyte-to-eosinophil ratio (MER), and lymphocyte-to-eosinophil ratio (LER).

Statistical analyses were performed using IBM SPSS Statistics version 26 (IBM Corp., Armonk, NY, USA). The normality of the data distribution was assessed using the Shapiro–Wilk and Kolmogorov–Smirnov tests. As the data were not normally distributed, the Kruskal-Wallis test was used to compare the continuous variables across the TT, EO, and control groups. Pairwise comparisons were performed using the Dunn-Bonferroni post-hoc test. Spearman’s rank correlation analysis was used to evaluate relationships between GPR and other continuous variables, including age and inflammatory markers. Receiver operating characteristic (ROC) curve analysis was conducted to evaluate the diagnostic performance of GPR and other indices in distinguishing TT. A combined model was constructed to assess the cumulative diagnostic accuracy, using the predicted probabilities derived from binary logistic regression (incorporating age, GPR, and WBC). The area under the curve (AUC), as well as the sensitivity and specificity, were calculated for each parameter. The optimal cut-off values were determined using the Youden index. To identify independent predictors of testicular torsion, a multivariate binary logistic regression analysis was performed using the Enter method. The multivariate model included the statistically significant variables identified in the univariate analysis. Odds ratios (OR) with 95% confidence intervals (CI) were calculated, and a p-value of less than 0.05 was considered statistically significant. Sample size estimation was based on a similar study, requiring at least 35 patients per group to achieve 80% power with a 5% margin of error [14].

## Results

The study population consisted of 373 participants, who were divided into three groups: the TT group (*n* = 103), the EO group (*n* = 120), and the control group (*n* = 150). Within the TT group, torsion was located on the left side in 64 patients and on the right side in 39 patients. In terms of surgical outcomes, detorsion was performed on 63 patients (61.2%), while orchiectomy was required due to testicular necrosis in 40 patients (38.8%). The demographic characteristics and laboratory parameters of these groups are summarised in Table 1. The EO group had a significantly higher median age (58 years) than the TT (18 years) and control (22 years) groups (*p* < 0.001).

**Table 1:** Demographic and laboratory characteristics of the testicular torsion, epididymorchitis, and control groups

	Testicular torsion (*n* = 103)	Epididymorchitis (*n* = 120)	Control group (*n* = 150)	*p* value*
Age (years)	18 (2–37)ᵃ	58 (10–86)ᵇ	22 (5–32)ᵃ	< 0.001
GPR	23.6 (15.2–68.3)ᵃ	21.0 (14.6–42.4)ᵇ	20.4 (13.1–35.7)ᶜ	< 0.001
WBC (10⁹ /L)	10.1 (5-33.3)ᵃ	10.1 (4.4–28.4)ᵃ	7 (4-20.3)ᵇ	< 0.001
Neutrophil count (10⁹ /L)	6.8 (1.84–30.7)ᵃ	6.4 (2.2–24.7)ᵃ	4.0 (1.3–13.6)ᵇ	< 0.001
Hematocrit (%)	42.3 (28-83.3)ᵃ	43.5 (31.4–51.8)ᵃ	46.1 (34.3–78.5)ᵇ	< 0.001
Platelet (10 ⁹ /L)	286 (131–730)ᵃ	246 (133–426)ᵇ	236 (138–653)ᵇ	< 0.001
MPV	9.3 (8-18.9)ᵃ	9.5 (7.7–12.8)ᵇ	9.8 (7.6–18.5)ᵇ	< 0.001
PDW	16.1 (15.1–32.1)ᵃ	16.2 (15.3–17.4)ᵇ	16.2 (15.2–31.8)ᵇ	< 0.001
SII	869 (101-15336)ᵃ	663.6 (199-13594)ᵃ	433.6 (131–3551)ᵇ	< 0.001
SIRI	2.16 (0.17–42.9)ᵃ	1.94 (0.34–43.7)ᵃ	0.8 (0.2–3.6)ᵇ	< 0.001
AISI	573 (40.6-23158)ᵃ	479.2 (77-10135)ᵃ	188.6 (30-2343)ᵇ	< 0.001
NLR	3.4 (0.4–28.4)ᵃ	2.9 (0.9–34)ᵃ	1.7 (0.6–5.6)ᵇ	< 0.001
PLR	139 (50–499)ᵃ	144.8 (48.6–1217)ᵃ	106 (48.5–260)ᵇ	< 0.001
MLR	0.25 (0.1–0.9)ᵃ	0.28 (0.11–2.82)ᵃ	0.19 (0.1–0.4)ᵇ	< 0.001
NER	37.9 (6.3-1229.5)ᵃ	29.6 (5.5–1853)ᵇ	24.2 (5-236.5)ᵇ	< 0.001
MER	4 (0.6–96)ᵃ	2.8 (0.7–163)ᵇ	2.8 (0.3–19.5)ᵇ	< 0.001
LER	17.7 (3.8–257)ᵃ	9.7 (2.38–253.6)ᵇ	15.3 (1.4–96.5)ᵃ	< 0.001

Data given as median (min-max), number. Different superscript letters (a, b, c) in the same row indicate statistically significant differences between groups (post-hoc analysis). *p*-values < 0.05 were considered statistically significant. * The Kruskal–Wallis test was used to compare the three groups

*Abbreviations*: *GPR* Glucose/potassium ratio, *WBC* White blood cell, *MPV* Mean platelet volume, *PDW* Platelet distribution width, *SII* Systemic inflammation index, *SIRI* Systemic inflammatory response index, *AISI* Aggregated index of systemic inflammation, *NLR* Neutrophil-to-lymphocyte ratio, *PLR* Platelet-to-lymphocyte ratio, *MLR* Monocyte-to-lymphocyte ratio, *NER* Neutrophil-to-eosinophil ratio, *MER* Monocyte-to-eosinophil ratio, *LER* lymphocyte-to-eosinophil ratio

When haematological and biochemical parameters were evaluated, the GPR was highest in the TT group (median: 23.6), followed by the EO group (median: 21.0) and the control group (median: 20.4) (*p* < 0.001). Pairwise comparisons revealed statistically significant differences in GPR levels between all three groups. Additionally, inflammatory markers, including white blood cell (WBC) count, neutrophil count, SII, SIRI, AISI, NLR, PLR, and MLR, were significantly higher in both the TT and EO groups than in the control group (*p* < 0.001). However, no significant difference was observed between the TT and EO groups for these markers. In contrast, the novel eosinophil-derived indices (NER, MER, LER) were significantly higher in the TT group than in the EO and control groups.

A Spearman correlation analysis was conducted to evaluate the relationships between the different parameters. No significant correlation was found between GPR and age (*r* = − 0.026, *p* = 0.663), indicating that GPR is an age-independent marker. Although GPR showed statistically significant correlations with other inflammatory markers (WBC, AISI, and NER), the correlation coefficients remained weak (*r* < 0.20). This suggests that GPR may reflect distinct pathophysiological processes, such as ischaemic stress, as well as inflammation.

**Fig. 2:**
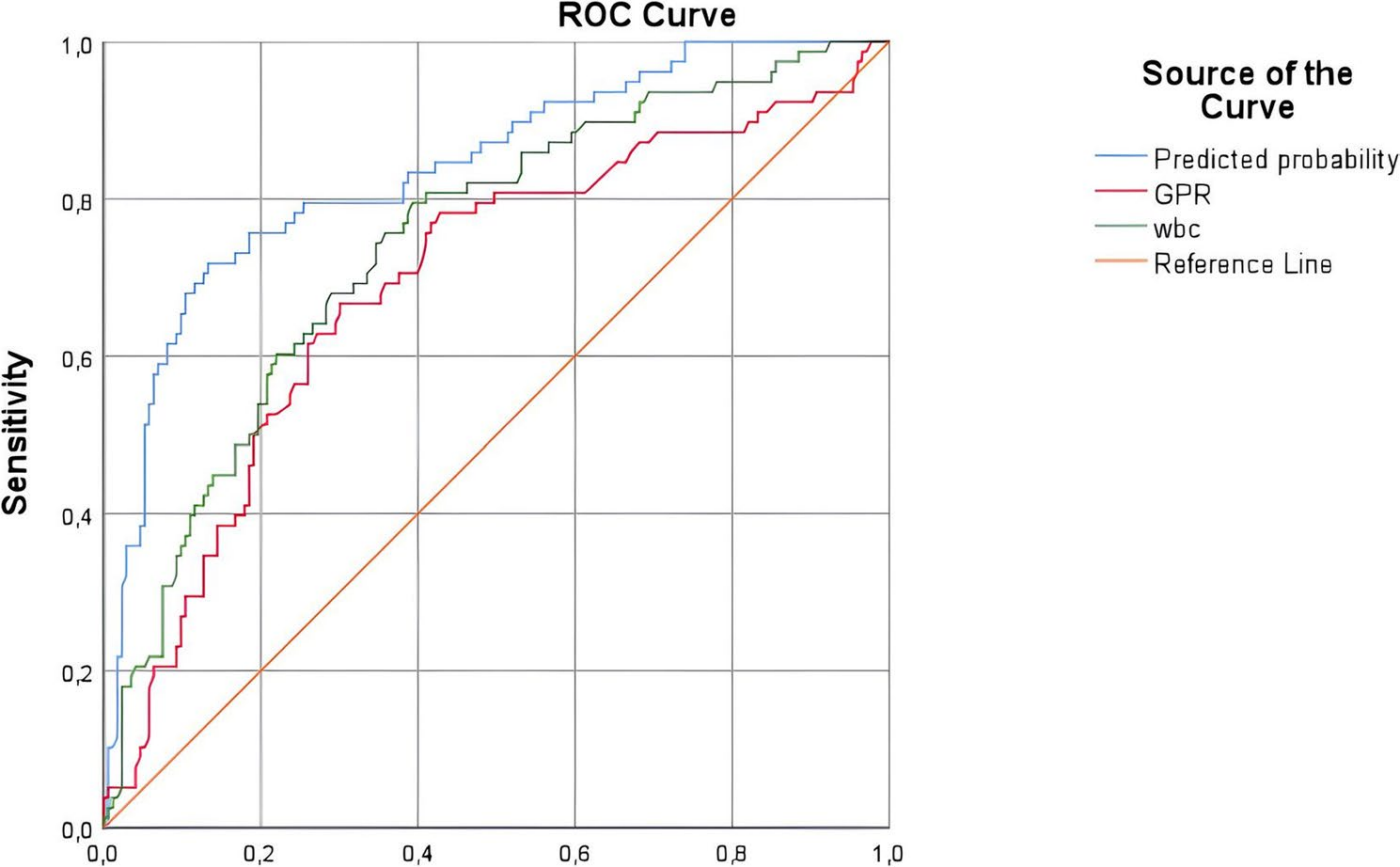
ROC curve analysis of the combined model, GPR, and WBC count in predicting testicular torsion. (Legend: Comparison of diagnostic performance in predicting testicular torsion. The ‘Predicted probability’ curve (blue line) shows that the combined model (constructed using binary logistic regression with age, GPR, and WBC) has superior diagnostic accuracy. GPR: Glucose/Potassium Ratio; WBC: White Blood Cell Count; ROC: Receiver Operating Characteristic)

A ROC curve analysis was performed to evaluate the diagnostic performance of clinical variables in predicting testicular torsion (Table 2; Fig. 2). Among all evaluated parameters, the combined model (incorporating age, GPR, and WBC) demonstrated superior diagnostic accuracy, with an AUC of 0.839 (95% CI: 0.784–0.893, *p* < 0.001). The model achieved a sensitivity of 75.6%, a specificity of 81.5%, a positive predictive value (PPV) of 60.9%, and a negative predictive value (NPV) of 89.7%. Of the individual clinical variables, GPR showed the highest performance, with an AUC of 0.738 (95% CI: 0.679–0.796, *p* < 0.001). With a cut-off value of 21.8, GPR demonstrated a sensitivity of 66.7%, a specificity of 65.0%, a PPV of 42.1%, and a NPV of 83.7%. Other significant predictors included WBC (AUC = 0.695), AISI (AUC = 0.666), SII (AUC = 0.649), NER (AUC = 0.638), and MER (AUC = 0.633).

**Table 2:** ROC analysis of the diagnostic performance of clinical variables in predicting testicular torsion

Variable	Cut-off	AUC	*p* value	95% CI	Sensitivity	Specificity
Combined Model	-	0.839	< 0.001	0.784–0.893	75.6%	81.5%
GPR	21.8	0.738	< 0.001	0.679–0.796	66.7%	65.0%
WBC	8.6	0.695	< 0.001	0.631–0.760	63.8%	63.0%
SII	596.1	0.649	< 0.001	0.572–0.725	63.0%	62.7%
SIRI	1.13	0.616	0.003	0.538–0.695	58.3%	55.1%
AISI	320.3	0.666	< 0.001	0.576–0.724	63.0%	62.0%
NLR	2.5	0.625	0.001	0.547–0.704	58.3%	62.6%
PLR	114.1	0.606	0.011	0.519–0.692	53.4%	53.6%
MER	3.4	0.633	0.001	0.555–0.711	61.6%	59.7%
NER	31.3	0.638	< 0.001	0.599–0.717	61.1%	60.3%

The data were analysed using a receiver operating characteristic (ROC) curve. The optimal cut-off values were determined based on the highest Youden index. The Combined Model was created using binary logistic regression probabilities, including Age, GPR, and WBC variables. *p*-values below 0.05 were considered statistically significant

*Abbreviations*: *AUC* Area under the curve, *CI* Confidence interval, *GPR* Glucose/potassium ratio, *WBC* White blood cell, *SII* Systemic inflammation index, *SIRI* Systemic inflammatory response index, *AISI *Aggregated index of systemic inflammation, *NLR* Neutrophil-to-lymphocyte ratio, *PLR* Platelet-to- lymphocyte ratio, *MER* Monocyte-to-eosinophil ratio, *NER* Neutrophil-to-eosinophil ratio

Following the assessment of general diagnostic performance, risk stratification for differential diagnosis between TT and EO (two conditions that are difficult to distinguish clinically) was evaluated (Table 3). Patients with a GPR value greater than 21.8 were 3.52 times more likely to be diagnosed with TT than those in the low-GPR group (OR: 3.52, 95% CI: 1.97–6.25; *p* < 0.001). Interestingly, while EO was the predominant diagnosis in the low-GPR group (approximately 72%), TT became the dominant diagnosis in the high-GPR group (approximately 58%). Although a GPR > 21.8 indicates a 3.52-fold increase in the risk of TT, it is important to note from a clinical perspective that a significant proportion (41.8%) of patients in this high-risk group presented with EO. This highlights the significant risk of false positives if GPR is evaluated in isolation.

**Table 3:** Risk analysis of the GPR cut-off value for differential diagnosis between testicular torsion and epididymorchitis

GPR Level	Testicular torsion, *n* (%)	Epididymorchitis, *n* (%)	OR (95% CI)	*p* value
High Risk (≥ 21.8)	78 (58.2%)	56 (41.8%)	3.52 (1.97–6.25)	< 0.001
Low Risk (< 21.8)	25 (28.1%)	64 (71.9%)	1 (Reference)	-

The cut-off value of 21.8 was determined by Youden’s index in the ROC analysis. *p*-values < 0.05 were considered statistically significant

*Abbreviations*: *OR* Odds ratio, *GPR* Glucose/Potassium Ratio, *CI* Confidence Interval

**Fig. 3:**
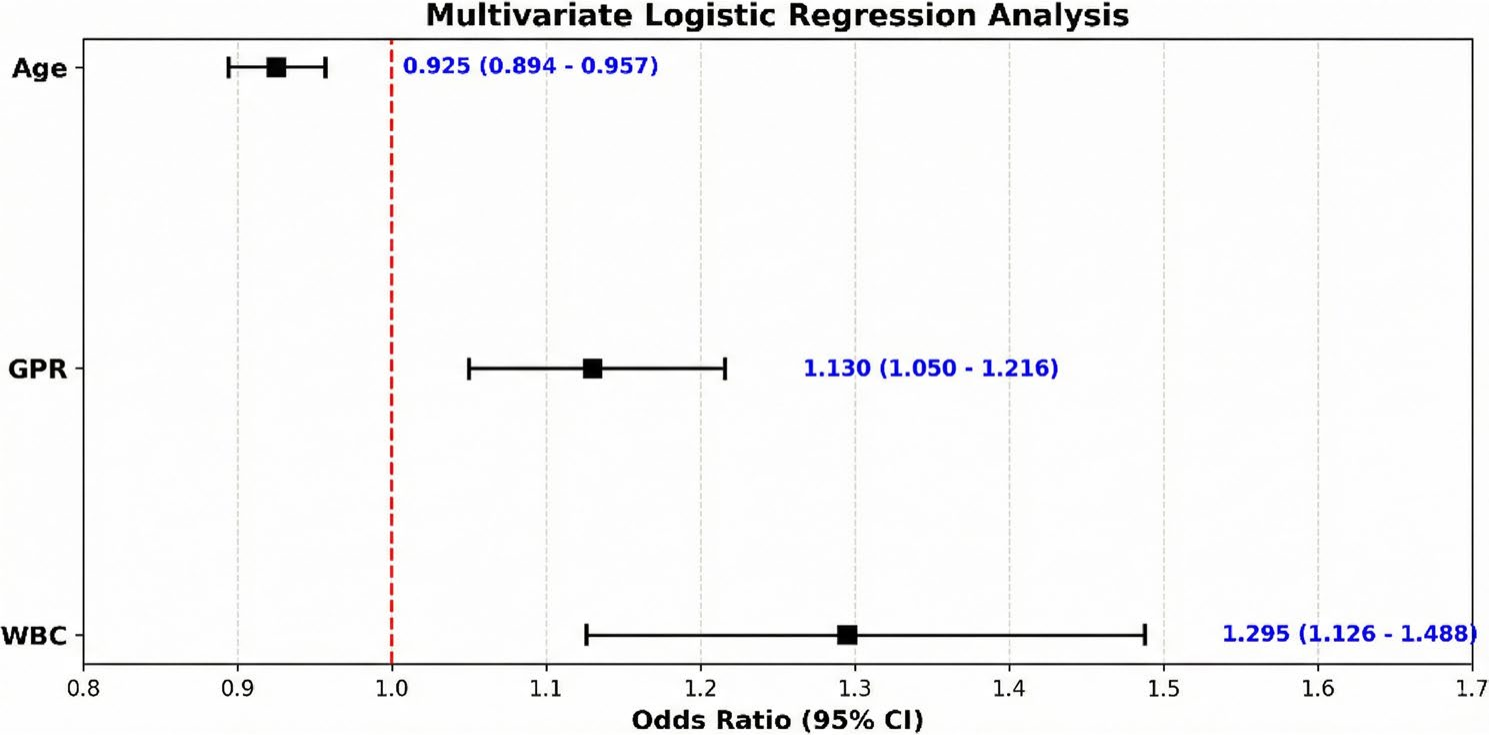
Forest plot illustrating the multivariate logistic regression analysis of independent predictors for testicular torsion. (Legend: This illustrates the multivariate logistic regression analysis. The squares represent the odds ratios (OR), and the horizontal lines represent the 95% confidence intervals (CI). GPR: glucose/potassium ratio; WBC: white blood cell count)

A multivariate logistic regression analysis was conducted to identify the independent predictors of TT (Table 4; Fig. 3). The analysis revealed that the following were independent predictors of TT: younger age (OR: 0.92, *p* < 0.001); higher WBC count (OR: 1.30, *p* < 0.001); higher GPR levels (OR: 1.14, 95% CI: 1.06–1.22, *p* = 0.001).

**Table 4:** A multivariate logistic regression analysis was performed to identify independent predictors for distinguishing testicular torsion

Variables	OR	95% CI	*p*-value
Age (years)	0.92	0.89–0.95	< 0.001
GPR	1.14	1.06–1.22	0.001
WBC	1.30	1.15–1.47	< 0.001

Multivariate logistic regression analysis was performed using the ‘Enter’ method. Due to high multicollinearity between WBC and inflammatory indices (such as AISI, SII), only WBC was included in the multivariate model as the representative inflammatory marker. A *p*-value of less than 0.05 was considered statistically significant

*Abbreviations*: *OR* Odds ratio, *CI* Confidence interval, *GPR* Glucose/Potassium Ratio, *WBC* White Blood Cell

## Discussion

To our knowledge, this is the first study to investigate the diagnostic value of the GPR in distinguishing TT from EO and non-specific scrotal pain. Furthermore, this study aims to develop metabolic profiling as a practical clinical tool. While previous research has predominantly focused on generic inflammatory markers, our study positions the GPR as a potential objective indicator of ischaemic stress. In real-world clinical practice, CDUS is the gold standard, but it is not always available in out-of-hours emergency settings, and the results can be operator-dependent or equivocal. In such scenarios, our combined model, incorporating age, WBC, and GPR, could serve as a rapid, ubiquitous, and low-cost adjunctive triage tool. Using routinely collected blood parameters, it could provide clinicians with objective risk stratification, potentially helping to prioritise urgent surgical evaluation for high-risk patients and minimising diagnostic uncertainty.

Acute scrotal pain is a common symptom associated with TT, EO, and a number of rarer conditions [15]. TT is a true surgical emergency characterised by twisting of the spermatic cord, which interrupts blood flow and progresses to ischaemia and necrosis within hours. Delayed diagnosis invariably results in testicular loss and infertility [16]. In contrast, EO and other causes of acute scrotal pain are predominantly managed conservatively with antibiotics and analgesics [17]. The main aim is to distinguish surgical emergencies, such as TT, from conditions that can be managed conservatively, such as EO, torsion of the appendix testis, and non-specific scrotal pain. A false-negative diagnosis (misdiagnosing TT) can result in orchiectomy, long-term hormonal impairment, and subfertility [18], whereas a false-positive diagnosis (misidentifying EO as TT) can expose patients to the risks of unnecessary surgical exploration and anaesthesia [19]. CDUS is the primary imaging modality for acute scrotal conditions and can differentiate between TT, EO, varicocele, hernia, and tumours with high diagnostic accuracy [20]. However, false-negative results may occur in early-stage cases or partial TT, where blood flow is preserved. Furthermore, its diagnostic performance remains operator-dependent [21]. Therefore, significant clinical value is attributed to objective biomarkers that can reliably differentiate these pathologies.

Rapid diagnosis of TT is crucial, as evaluation delays significantly increase orchiectomy rates [22]. Interestingly, prolonged ischaemic time has also been directly correlated with an increased WBC count at presentation [22], which further validates the inclusion of WBC alongside GPR in our combined diagnostic model. While testicular torsion is a time-critical condition, relying solely on CDUS can delay surgical exploration by up to two hours, thereby exacerbating the risk of testicular loss [23, 24]. Consequently, current studies emphasise immediate surgery without imaging for cases where torsion is highly suspected [25]. Although CDUS remains useful for equivocal cases, delayed access to ultrasound can be detrimental. As blood sampling occurs concurrently during initial triage, awaiting GPR and WBC results does not introduce sequential delays. In equivocal cases where CDUS is delayed or unavailable, our combined model could serve as a rapid, objective adjunctive tool for risk stratification, potentially accelerating the decision to perform surgery.

In the context of differential diagnosis, traditional inflammatory markers often prove inadequate. Several studies have shown that WBC levels, NLR, SII, and SIRI are significantly higher in cases of TT and EO than in healthy controls [26–28]. A meta-analysis found that leukocyte levels and NLR were higher in patients with TT than in healthy controls [29]. While some studies have reported higher values for indices such as NLR, PLR, SII, and SIRI in the TT group than in the EO group, ROC analyses generally indicate only weak-to-moderate diagnostic accuracy (AUC ~ 0.56–0.60) [28, 30]. In our study, consistent with previous research, we found that inflammatory indices, including WBC, neutrophil count, SII, SIRI, AISI, NLR, and PLR, were significantly higher in both the TT and EO groups than in the control group (non-specific pain). However, we observed no statistically significant difference in these parameters between the TT and EO groups. This finding highlights a significant limitation: while these markers can effectively indicate the presence of pathology, they lack the specificity to distinguish ischaemic necrosis in TT from infectious inflammation in EO. Additionally, a limited number of studies have shown that eosinophil-derived markers (MER) are elevated in cases of torsion and epididymitis [26]. However, in our study, the diagnostic accuracy of MER (AUC ~ 0.63) remained lower than that of GPR.

GPR has been utilized as an independent predictor of early mortality and poor prognosis in numerous critical ischaemic conditions, such as acute ischaemic stroke [31, 32], myocardial infarction [33], aortic dissection [34], intracranial hemorrhage [35], traumatic brain injury [36], spinal cord injury [37], and pulmonary embolism [38]. High GPR values facilitate rapid risk stratification and treatment prioritization, particularly in time-critical conditions such as acute ischaemic stroke and aortic dissection [31–34]. Ischaemia-reperfusion (I/R) injury following ischaemia creates a general stress response in the body, coupled with an increase in inflammatory cytokines (TNF-α, IL-6) and oxidative stress. This process can trigger the release of stress hormones via both the central nervous system and the endocrine system [39]. Following myocardial I/R injury, an increase in circulating catecholamine (adrenaline, noradrenaline) levels, concomitant with increased sympathetic nervous system activity, has been reported [40]. Furthermore, a marked increase in adrenocorticotropic hormone (ACTH) levels has been detected in experimental cerebral I/R models [41]. This subsequent rise in stress hormone levels leads to the elevation of glucose levels and the decrease of potassium levels. Therefore, we hypothesized that GPR could serve as a valuable tool for risk stratification in TT, a fundamentally ischaemic pathology.

This is where GPR demonstrates its unique diagnostic advantage. Unlike pure inflammatory markers, GPR reflects the acute metabolic stress response that is specific to severe ischaemia. Our results revealed a stepwise elevation in GPR levels, with the lowest levels recorded in the control group (median 20.4), intermediate levels recorded in the EO group (median 21.0), and significantly the highest levels recorded in the TT group (median 23.6). This gradient suggests that TT triggers a more profound sympathoadrenal surge and stress hyperglycaemia than the localised inflammation of EO or the benign course of non-specific pain [40].

However, the most clinically significant finding of our study was the superior performance of the combined model. Although GPR demonstrated the highest diagnostic accuracy among the individual parameters (AUC: 0.738), its sensitivity was only 66.7%. This indicates that it should not be used as a standalone screening tool. To overcome this limitation, we developed a combined model that integrates age, GPR, and WBC. This model achieved the highest diagnostic performance, with an AUC of 0.839, a sensitivity of 75.6%, and a specificity of 81.5%. This improvement is likely due to the synergistic evaluation of three distinct pathophysiological domains: Age (reflecting the demographic predisposition of TT in younger adolescents versus EO in older adults), WBC (reflecting general inflammation), and GPR (reflecting specific metabolic stress due to ischaemia). This multimodal approach may help reduce the risk of misdiagnosis compared to using any single marker in isolation.

In our study, the significant age difference between the groups reflects the natural epidemiology of these conditions. Notably, the diagnostic value of GPR remains independent of this demographic factor. GPR showed no correlation with age (*r* = − 0.026, *p* = 0.663) and was identified as an independent predictor of TT in the multivariate logistic regression analysis (OR: 1.14, 95% CI: 1.06–1.22, *p* = 0.001). These findings suggest that GPR may provide unique diagnostic insights based on ischaemic stress that are entirely independent of patient age and WBC count, rather than merely reflecting demographic or general inflammatory differences.

For clinicians, a GPR level exceeding 21.8 in a patient presenting with acute scrotal pain may indicate a significantly higher likelihood of torsion, with an increased risk of 3.52-fold. However, as 41.8% of patients in this group were diagnosed with EO, there is an inherent risk of false positives resulting in unnecessary surgical exploration. Therefore, this finding should strictly trigger an expedited CDUS evaluation rather than immediate surgery. Conversely, lower GPR levels might support a clinical decision towards a conservative approach, particularly when considered alongside clinical and sonographic findings. Moreover, GPR can be readily calculated from routine biochemical tests without additional cost, and it may serve as a rapid and practical biomarker, potentially making it suitable for risk stratification in emergency settings [31, 32].

## Limitations of the study

This study has certain limitations. Firstly, the retrospective design may introduce selection bias, and its single-centre nature means our proposed combined model currently lacks external validation, limiting the findings to a specific population. Secondly, serum glucose levels are affected by fasting status, which cannot be standardised in acute emergency settings. While ‘stress hyperglycaemia’ triggered by severe ischaemic pain likely overrides these nutritional variations, unstandardised fasting status remains an unavoidable confounding factor. Thirdly, due to the retrospective emergency nature of the study, detailed histories of specific medication use that might transiently influence glucose or potassium levels could not be fully standardised or evaluated. Finally, as CDUS was used as the reference standard to confirm the final diagnoses in our retrospective cohort, it was not possible to perform a direct, head-to-head prospective comparison of diagnostic accuracy between our combined model and CDUS. Therefore, our model should be considered an adjunctive triage tool and its findings hypothesis-generating, rather than a definitive replacement for sonographic evaluation.

## Conclusions

In conclusion, our study suggests that the novel metabolic biomarker GPR may offer distinct diagnostic value in reflecting ischaemic stress, potentially outperforming traditional inflammatory markers in distinguishing TT from EO. The combined model, which integrates age, GPR, and white blood cell count, shows preliminary promise in improving diagnostic accuracy and could serve as an adjunctive triage tool in emergency settings. However, as these findings are derived from a single-centre retrospective cohort, they remain hypothesis-generating. Future multicentre prospective studies with independent validation cohorts are required to validate the GPR cut-off value of 21.8, assess dynamic changes in GPR following detorsion, and verify the robustness of the combined model before its routine clinical implementation.

## Data Availability

The datasets used and/or analysed during the current study are available from the corresponding author on reasonable request.

## Abbreviations

ACTH: Adrenocorticotropic hormone
AISI: Aggregated index of systemic inflammation
AUC: Area under the curve
CBC: Complete blood count
CDUS: Colour Doppler Ultrasonography
CI: Confidence interval
EO: Epididymorchitis
GPR: Glucose/potassium ratio
I/R: Ischaemia-reperfusion
K₂EDTA: Dipotassium ethylenediaminetetraacetic acid
LER: Lymphocyte-to-eosinophil ratio
MER: Monocyte-to-eosinophil ratio
MLR: Monocyte-to-lymphocyte ratio
MPV: Mean platelet volume
NER: Neutrophil-to-eosinophil ratio
NLR: Neutrophil-to-lymphocyte ratio
NPV: Negative predictive value
OR: Odds ratio
PDW: Platelet distribution width
PLR: Platelet-to-lymphocyte ratio
PPV: Positive predictive value
ROC: Receiver Operating Characteristic
SII: Systemic immune-inflammation index
SIRI: Systemic inflammation response index
TT: Testicular torsion
WBC: White blood cell
